# Charting human organogenesis across the Carnegie stages from a whole-embryo perspective

**DOI:** 10.1093/gigascience/giag063

**Published:** 2026-05-28

**Authors:** Jiexue Pan, Zhongliang Lin, Gaochen Zhang, Hefeng Huang, Guolian Ding

**Affiliations:** Institute of Reproduction and Development, Shanghai Key Laboratory of Reproduction and Development, Obstetrics and Gynecology Hospital, Fudan University, Shanghai 200011, China; Shanghai Key Laboratory of Female Reproductive Endocrine Related Diseases, Obstetrics and Gynecology Hospital, Fudan University, Shanghai 200011, China; Institute of Medical Genetics and Development, Key Laboratory of Reproductive Genetics (Ministry of Education) and Women’s Hospital, Zhejiang University School of Medicine, Hangzhou 310058, China; Department of Obstetrics and Gynecology, Center for Reproductive Medicine, the Fourth Affiliated Hospital of School of Medicine, and International School of Medicine, International Institutes of Medicine, Zhejiang University, Yiwu 322099, China; Institute of Reproduction and Development, Shanghai Key Laboratory of Reproduction and Development, Obstetrics and Gynecology Hospital, Fudan University, Shanghai 200011, China; Shanghai Key Laboratory of Female Reproductive Endocrine Related Diseases, Obstetrics and Gynecology Hospital, Fudan University, Shanghai 200011, China; Institute of Reproduction and Development, Shanghai Key Laboratory of Reproduction and Development, Obstetrics and Gynecology Hospital, Fudan University, Shanghai 200011, China; Institute of Medical Genetics and Development, Key Laboratory of Reproductive Genetics (Ministry of Education) and Women’s Hospital, Zhejiang University School of Medicine, Hangzhou 310058, China; Department of Obstetrics and Gynecology, Center for Reproductive Medicine, the Fourth Affiliated Hospital of School of Medicine, and International School of Medicine, International Institutes of Medicine, Zhejiang University, Yiwu 322099, China; Institute of Reproduction and Development, Shanghai Key Laboratory of Reproduction and Development, Obstetrics and Gynecology Hospital, Fudan University, Shanghai 200011, China; Shanghai Key Laboratory of Female Reproductive Endocrine Related Diseases, Obstetrics and Gynecology Hospital, Fudan University, Shanghai 200011, China

**Keywords:** human development, spatial transcriptomics, organogenesis, Human Developmental Cell Atlas, Carnegie stages

## Abstract

The Carnegie stages represent a critical window in human development, during which organ primordia emerge, tissue identities diversify, and many congenital disorders are thought to originate. However, this period has remained difficult to study at the whole-embryo scale. Recent advances in spatial and single-cell genomics are beginning to close this gap. This article discusses the significance of the spatiotemporal transcriptomic atlas of post-gastrulation human embryos spanning Carnegie stages 12–23, which provides one of the most comprehensive dynamic molecular maps of this developmental window to date. Beyond its value as a reference resource, this atlas offers biological insights into early cardiac patterning, brain regionalization, the timing of inhibitory and excitatory neurogenesis, tissue-specific susceptibility to prenatal infection, and spatially resolved allelic imbalance. Equally importantly, it illustrates a broader shift in the field from fragmented organ-specific datasets toward integrated developmental frameworks that can connect embryology, human genetics, and disease mechanisms. Future progress will depend not only on generating more data but also on harmonizing multimodal datasets across sources, stages, and platforms, an effort that will increasingly rely on advances in computational biology and artificial intelligence.

## Why the Carnegie stages matter

The period following gastrulation is one of the most important but least accessible phases of human development. During the Carnegie stages, the embryo rapidly becomes more complex, and major organs begin to emerge and acquire distinct identities. This is also a period of marked developmental vulnerability. Many congenital anomalies and neurodevelopmental disorders are thought to originate during these weeks, which may also represent a window in which maternal infection is more likely to cause severe adverse embryonic outcomes. Despite its importance, this interval has remained difficult to characterize at whole-embryo resolution, partly because it cannot be studied *in vitro* in the same way as earlier human embryos and because it does not permit the relatively high-resolution imaging that becomes possible during the fetal period. Earlier work established important single-cell atlases of fetal tissue [1] and early organogenesis [2], while spatial studies clarified aspects of gastrulation [3] and selected organs [4]. However, no study had comprehensively captured post-gastrulation human development across the Carnegie stages in a dynamic, spatially resolved, whole-embryo manner.

This gap concerns not only coverage but also interpretation. Organogenesis is a spatially coordinated process, yet much of the existing evidence has come either from dissociated single cells, in which anatomical context is lost, or from spatial studies restricted to limited stages or individual tissues. As a result, the field has accumulated many informative datasets, but these remain difficult to assemble into a coherent account of whole-embryo development. The Carnegie stages are therefore especially important because they mark the interval during which cellular diversification is translated into anatomical organization and early organ-specific function.

## From atlas to mechanism

The recent spatiotemporal atlas of human embryos spanning Carnegie stages 12–23 aims to fill this gap [5]. By integrating whole-embryo Stereo-seq [6] with single-nucleus RNA sequencing, the study resolved 50 organs or anatomical regions and 198 substructures across the critical interval from ~4 to 8 weeks after conception. In doing so, it provides the most comprehensive dynamic atlas yet of human embryogenesis after gastrulation across the Carnegie stages. Its importance, however, lies not only in its scale but also in the biological questions that this scale makes possible. A whole-embryo atlas does more than increase the number of data points. It restores developmental context and enables organogenesis to be examined as a coordinated process rather than as a set of disconnected organ-specific events [5].

This context is essential because many developmental mechanisms cannot be understood independently of anatomy. In the heart, for example, spatial resolution enabled fine cardiac substructures to be distinguished and their associated regulatory programs to be reconstructed. Chamber-specific transcriptional patterns became clearer, and the embryonic sinoatrial node could be localized and analyzed at molecular resolution. Regulators such as *RORA* and *KIAA1324L* emerged as candidate factors in pacemaker development, and these findings were not left as purely computational predictions. Their relevance was supported by external datasets and by *in vivo* functional assays. A similar principle applies to the developing brain. Fine regionalization could be mapped unusually early, and the timing of neuronal lineage emergence could be reassessed with stronger anatomical grounding. Inhibitory neuron markers were detectable as early as Carnegie stages 12–13 in the ganglionic eminence, whereas excitatory neuron specification appeared later in the pallium, refining the current timeline of early human neurogenesis [7]. This was not merely a matter of annotation. HMGA2-centered regulatory programs were linked to pallial development and to genes implicated in intellectual disability, and the observations were strengthened by external validation and complementary single-nucleus data [5].

## Developmental vulnerability in a clinical context

The atlas is also relevant to prenatal medicine. One longstanding clinical question is why maternal infection during early pregnancy can produce severe structural or functional abnormalities at some developmental stages but not at others. Clinical decision-making in this setting has often been guided more by epidemiology and clinical experience than by direct embryonic evidence. By mapping the spatial and temporal expression of host factors used by pathogens associated with congenital disease, including cytomegalovirus, Zika virus, hepatitis B virus, and SARS-CoV-2, the atlas provides a molecular framework for understanding developmental susceptibility. These receptor patterns are highly organ- and stage-specific, helping explain why certain embryonic tissues may be especially vulnerable during narrow developmental windows. This is particularly important in obstetrics, where management after early gestational infection is still often based on incomplete mechanistic knowledge.

Another important contribution concerns allelic imbalance during organogenesis. By resolving the spatial expression of known imprinted genes and identifying organ-specific imbalance in non-imprinted genes, the data open a new window on parent-of-origin effects in early human development. These observations may help explain why some developmental disorders show tissue specificity or sex-biased features, while also providing a basis for identifying additional candidate imprinted genes. This moves the discussion beyond cell identity toward a more layered understanding of embryonic regulation, one that includes gene dosage, parental origin, and tissue context.

Together, these examples suggest that the most important contribution of a whole-embryo atlas may not be its descriptive completeness alone. Rather, its value lies in allowing developmental vulnerability to be studied as a spatially organized property of the embryo. This represents a conceptual shift. It brings embryology closer to mechanism and brings mechanism closer to clinical interpretation. In the future, moving from correlation to causality will require active validation using corresponding models, including organoids and animal systems.

## The next bottleneck is not data generation but data integration

This work should also be considered in the broader context of developmental atlas building. Recent reviews have made clear that the field is entering a new phase. The main challenge is no longer simply how to generate more single-cell or spatial datasets but how to integrate them. Human developmental data now come from different organs, platforms, sampling strategies, sequencing depths, annotation systems, and gestational windows. The result is a growing abundance of information, but not yet a fully coherent developmental framework.

For this reason, one of the most important future directions is the normalization and harmonization of data across sources. Whole-embryo spatial maps, organ-resolved single-cell atlases, epigenomic profiles, lineage information, and disease-associated datasets will become far more valuable once they can be systematically aligned. This is not a trivial computational task. It requires reliable cross-platform registration, stage-aware batch correction, anatomically informed cell-state matching, and principled approaches to uncertainty. In prenatal development, even small differences in stage assignment or tissue sampling can alter interpretation. Data integration in this field therefore cannot simply be borrowed from adult tissue atlas studies. It will require methods explicitly designed for developmental trajectories, transient cell states, and shifting anatomical boundaries.

Computational biology and, increasingly, artificial intelligence (AI) may become transformative in this context. AI is unlikely to replace embryology, but it may substantially improve how developmental data are integrated, annotated, and interpreted. Foundation-model-style approaches, graph-based alignment methods, and multimodal representation learning could help connect datasets generated from different platforms and cohorts. They may also improve the inference of missing modalities, temporal ordering, and cross-species comparisons. If applied carefully, such approaches could support the transition from isolated maps to interoperable developmental reference systems. However, this will require high-quality training data, careful benchmarking, and continued biological validation. In developmental biology, algorithmic sophistication alone is not sufficient. Interpretability and anatomical fidelity remain essential.

The recent Human Developmental Cell Atlas (HDCA) preprint points toward this future [8]. By harmonizing cross-organ prenatal datasets and combining them with spatially defined tissue niches, it shows how developmental atlases can become more integrated and more clinically useful. The lesson is not that one atlas will replace another, but that the field is moving toward a layered ecosystem of datasets. Whole-embryo spatial atlases will define broad developmental context. Organ-specific single-cell atlases will add cellular depth. Multi-omics and perturbational datasets will provide regulatory mechanisms. Clinical and genetic datasets will connect those maps to disease. The challenge now is to make these layers interoperable. Addressing this challenge will demand not only algorithmic innovation but also data standards and ethical frameworks.

## Conclusions

### What comes next for human developmental atlases

Several directions are now especially important. First, future atlases should move beyond transcriptomics alone and integrate epigenomic, metabolic, proteomic, and lineage-level information, allowing developmental states to be interpreted mechanistically. Second, the field will benefit from more explicit three-dimensional and longitudinal reconstruction so that development can be followed as a continuous process rather than inferred from sectional snapshots. Third, integration with clinical genetics should become a priority. Developmental atlases are increasingly able to identify where and when disease genes are active, but the next challenge is to connect those patterns rigorously to pathological mechanisms and clinical phenotypes. Finally, species differences remain substantial, especially in the nervous system. Human data will therefore remain indispensable, even as organoid systems and model organisms continue to provide experimental depth [9, 10].

A whole-embryo atlas of post-gastrulation human development does more than add another resource to the field. It changes the scale at which organogenesis can be studied and, in doing so, changes the kinds of questions that can be asked. The next step will not be simply to build larger atlases. It will be to build more connected ones. If this is achieved, developmental biology may begin to link spatial organization, regulatory mechanisms, and disease origin within a single interpretive framework. The ultimate goal is not a static map but a predictive, dynamic model of human development.

## Abbreviation

HDCA: Human Developmental Cell Atlas.

## Supplementary Material

giag063_GIGA-D-26-00150_original_submission

## Data Availability

Not applicable.
