# Supplementary material for: Charting human organogenesis across the Carnegie stages from a whole-embryo perspective
Source: Gigascience. 2026 May 28;15:giag063. doi: 10.1093/gigascience/giag063 (PMC13231842; doi:10.1093/gigascience/giag063)
Supplement: giag063_GIGA-D-26-00150_original_submission [file giag063_giga-d-26-00150_original_submission.pdf]

GigaScience

Charting human organogenesis across the Carnegie stages from a whole-embryo perspective

--Manuscript Draft--

**Manuscript Number:**	GIGA-D-26-00150
**Full Title:**	Charting human organogenesis across the Carnegie stages from a whole-embryo perspective
**Article Type:**	Commentary
**Funding Information:**	
**Abstract:**	The Carnegie stages represent a critical window in human development, during which organ primordia emerge, tissue identities diversify, and many congenital disorders are thought to originate. Yet this period has remained difficult to study at whole-embryo scale. Recent advances in spatial and single-cell genomics are beginning to close this gap. Here, we discuss the significance of the spatiotemporal transcriptomic atlas of post-gastrulation human embryos spanning Carnegie stages 12–23, which provides, to our knowledge, the most comprehensive dynamic molecular map of this developmental window to date. Beyond its value as a reference resource, this atlas offers biological insights into early cardiac patterning, brain regionalization, the timing of inhibitory and excitatory neurogenesis, tissue-specific susceptibility to prenatal infection, and spatially resolved allelic imbalance. Just as importantly, it illustrates a broader shift in the field, from fragmented organ-specific datasets toward integrated developmental frameworks that can connect embryology, human genetics, and disease mechanisms. Future progress will depend not only on generating more data, but also on harmonizing multimodal datasets across sources, stages, and platforms, an effort that will increasingly rely on advances in computational biology and artificial intelligence.
**Corresponding Author:**	Guolian Ding, M.D. & Ph.D.<br>Fudan University<br>Shanghai, Shanghai CHINA
**Corresponding Author Secondary Information:**	
**Corresponding Author's Institution:**	Fudan University
**Corresponding Author's Secondary Institution:**	
**First Author:**	Jiexue Pan
**First Author Secondary Information:**	
**Order of Authors:**	Jiexue Pan
	Zhongliang Lin
	Gaochen Zhang
	Hefeng Huang
	Guolian Ding, M.D. & Ph.D.
**Order of Authors Secondary Information:**	
**Additional Information:**	
**Question**	**Response**
Are you submitting this manuscript to a special series or article collection?	No
**Experimental design and statistics**	No

Powered by Editorial Manager® and ProduXion Manager® from Aries Systems Corporation

Full details of the experimental design and statistical methods used should be given in the Methods section, as detailed in our Minimum Standards Reporting Checklist. Information essential to interpreting the data presented should be made available in the figure legends.<br><br>Have you included all the information requested in your manuscript?	
If not, please give reasons for any omissions below.<br><br>as follow-up to "**Experimental design and statistics**<br><br>Full details of the experimental design and statistical methods used should be given in the Methods section, as detailed in our Minimum Standards Reporting Checklist. Information essential to interpreting the data presented should be made available in the figure legends.<br><br>Have you included all the information requested in your manuscript?<br><br>"	This is a Perspective, not original article.
**Resources**<br><br>A description of all resources used, including antibodies, cell lines, animals and software tools, with enough information to allow them to be uniquely identified, should be included in the Methods section. Authors are strongly encouraged to cite Research Resource Identifiers (RRIDs) for antibodies, model organisms and tools, where possible.<br><br>Have you included the information requested as detailed in our Minimum Standards Reporting Checklist?	No

If not, please give reasons for any omissions below.<br><br>as follow-up to "**Resources**<br><br>A description of all resources used, including antibodies, cell lines, animals and software tools, with enough information to allow them to be uniquely identified, should be included in the Methods section. Authors are strongly encouraged to cite Research Resource Identifiers (RRIDs) for antibodies, model organisms and tools, where possible.<br><br>Have you included the information requested as detailed in our Minimum Standards Reporting Checklist?<br><br>"	This perspective does not generate original data and is not associated with experimental design.
**Availability of data and materials**<br><br>All datasets and code on which the conclusions of the paper rely must be either included in your submission or deposited in publicly available repositories (where available and ethically appropriate), referencing such data using a unique identifier in the references and in the "Availability of Data and Materials" section of your manuscript.<br><br>Have you have met the above requirement as detailed in our Minimum Standards Reporting Checklist?	No
If not, please give reasons for any omissions below.<br><br>as follow-up to "**Availability of data and materials**<br><br>All datasets and code on which the	This perspective does not generate original data and is not associated with experimental design.

Powered by Editorial Manager® and ProduXion Manager® from Aries Systems Corporation

conclusions of the paper rely must be either included in your submission or deposited in publicly available repositories (where available and ethically appropriate), referencing such data using a unique identifier in the references and in the "Availability of Data and Materials" section of your manuscript.<br><br>Have you have met the above requirement as detected in our Minimum Standards Reporting Checklist?<br><br>"	
GigaScience has policies and guidelines in place for the use of generative AI-writing tools such as ChatGPT. If you have used such writing tools to assist with writing the manuscript this must be declared and cited in the text. Authors should not list AI-writing tools and other AI-assisted technologies as an author or co-author and should acknowledge that they are fully responsible for text generated or refined by AI-writing tools.<p><br><br>A summary of use (particularly in the introduction or among methods) needs to be included at the end of the paper, and the outputs should also be included as a supplementary file hosted in GigaDB or other open repositories. Please read our guidelines for more information. <p><br><br>By submitting to GigaScience, you are aware of the journal's AI-writing tools policy, and if you have declared use of such tools below, you have acknowledged this where appropriate in your manuscript and have made a summary of use and outputs available. <p><br><b>AI-assisted writing tools have been used in the preparation of this manuscript?	No

Powered by Editorial Manager® and ProduXion Manager® from Aries Systems Corporation

Manuscript

Click here to access/download;Manuscript;Charting human organogenesis across the Carnegie clean.docx

Charting human organogenesis across the Carnegie stages from a whole-embryo perspective

Jiexue Pan (潘洁雪)[1,2,#,*], Zhongliang Lin (林忠亮)[3,4,#], Gaochen Zhang (张杲琛)[1,2], Hefeng Huang (黄荷凤)[1,3,4,*], Guolian Ding (丁国莲)[1,2,*]

Affiliations

[1] Institute of Reproduction and Development, Shanghai Key Laboratory of Reproduction and Development, Obstetrics and Gynecology Hospital, Fudan University, Shanghai, 200011, China

[2] Shanghai Key Laboratory of Female Reproductive Endocrine Related Diseases, Shanghai, China

[3] Institute of Medical Genetics and Development, Key Laboratory of Reproductive Genetics (Ministry of Education) and Women's Hospital, Zhejiang University School of Medicine, Hangzhou, 310058, China

[4] Department of Obstetrics and Gynecology, Center for Reproductive Medicine, the Fourth Affiliated Hospital of School of Medicine, and International School of Medicine, International Institutes of Medicine, Zhejiang University, Yiwu 322099, China

[#] These authors contributed equally to this work

*Correspondence to: dingguolian@fudan.edu.cn (G.D.); huanghefg@fudan.edu.cn (H.H.); panjiexue@fudan.edu.cn (J.P.)

ORCID:

Guolian Ding:0000-0002-0099-9980

Hefeng Huang: 0000-0002-0195-985X

Jiexue Pan: 0000-0001-7125-9608

Gaochen Zhang: 0009-0004-6122-7417

Zhongliang Lin: 0009-0000-8311-5193

Abstract
The Carnegie stages represent a critical window in human development, during which organ primordia emerge, tissue identities diversify, and many congenital disorders are thought to originate. However, this period has remained difficult to study at the whole-embryo scale. Recent advances in spatial and single-cell genomics are beginning to close this gap. This article discusses the significance of the spatiotemporal transcriptomic atlas of post-gastrulation human embryos spanning Carnegie stages 12–23, which provides one of the most comprehensive dynamic molecular maps of this developmental window to date. Beyond its value as a reference resource, this atlas offers biological insights into early cardiac patterning, brain regionalization, the timing of inhibitory and excitatory neurogenesis, tissue-specific susceptibility to prenatal infection, and spatially resolved allelic imbalance. Equally importantly, it illustrates a broader shift in the field from fragmented organ-specific datasets toward integrated developmental frameworks that can connect embryology, human genetics, and disease mechanisms. Future progress will depend not only on generating more data but also on harmonizing multimodal datasets across sources, stages, and platforms, an effort that will increasingly rely on advances in computational biology and artificial intelligence.

Why the Carnegie stages matter

The period following gastrulation is one of the most important but least accessible phases of human development. During the Carnegie stages, the embryo rapidly becomes more complex, and major organs begin to emerge and acquire distinct identities. This is also a period of marked developmental vulnerability. Many congenital anomalies and neurodevelopmental disorders are thought to originate during these weeks, which may also represent a window in which maternal infection is more likely to cause severe adverse embryonic outcomes. Despite its importance, this interval has remained difficult to characterize at whole-embryo resolution, partly because it cannot be studied *in vitro* in the same way as earlier human embryos and because it does not permit the relatively high-resolution imaging that becomes possible during the fetal period. Earlier work established important single-cell atlases of fetal tissue [1] and early organogenesis [2], while spatial studies clarified aspects of gastrulation [3] and selected organs [4]. However, no study had comprehensively captured post-gastrulation human development across the Carnegie stages in a dynamic, spatially resolved, whole-embryo manner.

This gap concerns not only coverage but also interpretation. Organogenesis is a spatially coordinated process, yet much of the existing evidence has come either from dissociated single cells, in which anatomical context is lost, or from spatial studies restricted to limited stages or individual tissues. As a result, the field has accumulated many informative datasets, but these remain difficult to

assemble into a coherent account of whole-embryo development. The Carnegie stages are therefore especially important because they mark the interval during which cellular diversification is translated into anatomical organization and early organ-specific function.

From atlas to mechanism

The recent spatiotemporal atlas of human embryos spanning Carnegie stages 12–23 aims to fill this gap [5]. By integrating whole-embryo Stereo-seq [6] with single-nucleus RNA sequencing, the study resolved 50 organs or anatomical regions and 198 substructures across the critical interval from approximately 4–8 weeks after conception. In doing so, it provides the most comprehensive dynamic atlas yet of human embryogenesis after gastrulation across the Carnegie stages. Its importance, however, lies not only in its scale but also in the biological questions that this scale makes possible. A whole-embryo atlas does more than increase the number of data points. It restores developmental context and enables organogenesis to be examined as a coordinated process rather than as a set of disconnected organ-specific events [5].

This context is essential because many developmental mechanisms cannot be understood independently of anatomy. In the heart, for example, spatial resolution enabled fine cardiac substructures to be distinguished and their associated regulatory programs to be reconstructed. Chamber-specific transcriptional patterns became clearer, and the embryonic sinoatrial node could be localized and analyzed at molecular resolution. Regulators such as *RORA* and *KIAA1324L* emerged as candidate factors in pacemaker development, and these findings were not left as purely computational predictions. Their relevance was supported by external datasets and by *in vivo* functional assays. A similar principle applies to the developing brain. Fine regionalization could be mapped unusually early, and the timing of neuronal lineage emergence could be reassessed with stronger anatomical grounding. Inhibitory neuron markers were detectable as early as Carnegie stages 12–13 in the ganglionic eminence, whereas excitatory neuron specification appeared later in the pallium, refining the current timeline of early human neurogenesis [7]. This was not merely a matter of annotation. HMGA2-centered regulatory programs were linked to pallial development and to genes implicated in intellectual disability, and the observations were strengthened by external validation and complementary single-nucleus data [5].

Developmental vulnerability in a clinical context

The atlas is also relevant to prenatal medicine. One longstanding clinical question is why maternal infection during early pregnancy can produce severe structural or functional abnormalities at some developmental stages but not at others. Clinical decision-making in this setting has often been guided more by

epidemiology and clinical experience than by direct embryonic evidence. By mapping the spatial and temporal expression of host factors used by pathogens associated with congenital disease, including cytomegalovirus, Zika virus, hepatitis B virus, and SARS-CoV-2, the atlas provides a molecular framework for understanding developmental susceptibility. These receptor patterns are highly organ- and stage-specific, helping explain why certain embryonic tissues may be especially vulnerable during narrow developmental windows. This is particularly important in obstetrics, where management after early gestational infection is still often based on incomplete mechanistic knowledge.

Another important contribution concerns allelic imbalance during organogenesis. By resolving the spatial expression of known imprinted genes and identifying organ-specific imbalance in non-imprinted genes, the data open a new window on parent-of-origin effects in early human development. These observations may help explain why some developmental disorders show tissue specificity or sex-biased features, while also providing a basis for identifying additional candidate imprinted genes. This moves the discussion beyond cell identity toward a more layered understanding of embryonic regulation, one that includes gene dosage, parental origin, and tissue context.

Together, these examples suggest that the most important contribution of a whole-embryo atlas may not be its descriptive completeness alone. Rather, its value lies in allowing developmental vulnerability to be studied as a spatially organized property of the embryo. This represents a conceptual shift. It brings embryology closer to mechanism and brings mechanism closer to clinical interpretation. In the future, moving from correlation to causality will require active validation using corresponding models, including organoids and animal systems.

The next bottleneck is not data generation but data integration

This work should also be considered in the broader context of developmental atlas building. Recent reviews have made clear that the field is entering a new phase. The main challenge is no longer simply how to generate more single-cell or spatial datasets but how to integrate them. Human developmental data now come from different organs, platforms, sampling strategies, sequencing depths, annotation systems, and gestational windows. The result is a growing abundance of information, but not yet a fully coherent developmental framework.

For this reason, one of the most important future directions is the normalization and harmonization of data across sources. Whole-embryo spatial maps, organ-resolved single-cell atlases, epigenomic profiles, lineage information, and disease-associated datasets will become far more valuable once they can

be systematically aligned. This is not a trivial computational task. It requires reliable cross-platform registration, stage-aware batch correction, anatomically informed cell-state matching, and principled approaches to uncertainty. In prenatal development, even small differences in stage assignment or tissue sampling can alter interpretation. Data integration in this field therefore cannot simply be borrowed from adult tissue atlas studies. It will require methods explicitly designed for developmental trajectories, transient cell states, and shifting anatomical boundaries.

Computational biology and, increasingly, artificial intelligence (AI) may become transformative in this context. AI is unlikely to replace embryology, but it may substantially improve how developmental data are integrated, annotated, and interpreted. Foundation-model-style approaches, graph-based alignment methods, and multimodal representation learning could help connect datasets generated from different platforms and cohorts. They may also improve the inference of missing modalities, temporal ordering, and cross-species comparisons. If applied carefully, such approaches could support the transition from isolated maps to interoperable developmental reference systems. However, this will require high-quality training data, careful benchmarking, and continued biological validation. In developmental biology, algorithmic sophistication alone is not sufficient. Interpretability and anatomical fidelity remain essential.

The recent Human Developmental Cell Atlas (HDCA) preprint points toward this future [8]. By harmonizing cross-organ prenatal datasets and combining them with spatially defined tissue niches, it shows how developmental atlases can become more integrated and more clinically useful. The lesson is not that one atlas will replace another, but that the field is moving toward a layered ecosystem of datasets. Whole-embryo spatial atlases will define broad developmental context. Organ-specific single-cell atlases will add cellular depth. Multi-omics and perturbational datasets will provide regulatory mechanisms. Clinical and genetic datasets will connect those maps to disease. The challenge now is to make these layers interoperable. Addressing this challenge will demand not only algorithmic innovation but also data standards and ethical frameworks.

Conclusions

What comes next for human developmental atlases

Several directions are now especially important. First, future atlases should move beyond transcriptomics alone and integrate epigenomic, metabolic, proteomic, and lineage-level information, allowing developmental states to be interpreted mechanistically. Second, the field will benefit from more explicit three-dimensional and longitudinal reconstruction so that development can be

followed as a continuous process rather than inferred from sectional snapshots. Third, integration with clinical genetics should become a priority. Developmental atlases are increasingly able to identify where and when disease genes are active, but the next challenge is to connect those patterns rigorously to pathological mechanisms and clinical phenotypes. Finally, species differences remain substantial, especially in the nervous system. Human data will therefore remain indispensable, even as organoid systems and model organisms continue to provide experimental depth [9, 10].

A whole-embryo atlas of post-gastrulation human development does more than add another resource to the field. It changes the scale at which organogenesis can be studied and, in doing so, changes the kinds of questions that can be asked. The next step will not be simply to build larger atlases. It will be to build more connected ones. If this is achieved, developmental biology may begin to link spatial organization, regulatory mechanisms, and disease origin within a single interpretive framework. The ultimate goal is not a static map but a predictive, dynamic model of human development.

Abbreviations

HDCA: Human Developmental Cell Atlas.

Data Availability

Not applicable.

Competing Interests

The author declares that they have no competing interests.

References

1. Cao J, O'Day DR, Pliner HA, Kingsley PD, Deng M, Daza RM, et al. A human cell atlas of fetal gene expression. Science. 2020;370 6518; doi:10.1126/science.aba7721.
2. Xu Y, Zhang T, Zhou Q, Hu M, Qi Y, Xue Y, et al. A single-cell transcriptome atlas profiles early organogenesis in human embryos. Nature Cell Biology. 2023;25 4:604-15; doi:10.1038/s41556-023-01108-w.
3. Cui L, Lin S, Yang X, Xie X, Wang X, He N, et al. Spatial transcriptomic characterization of a Carnegie stage 7 human embryo. Nature Cell Biology. 2025;27 2:360-9; doi:10.1038/s41556-024-01597-3.
4. Zeng B, Liu Z, Lu Y, Zhong S, Qin S, Huang L, et al. The single-cell and spatial transcriptional landscape of human gastrulation and early brain development. Cell Stem Cell. 2023;30 6:851-66.e7; doi:10.1016/j.stem.2023.04.016.
5. Pan J, Li Y, Lin Z, Lan Q, Zhang Y, Chen H, et al. Spatiotemporal transcriptome atlas of human embryos after gastrulation. Nature. 2026; doi:10.1038/s41586-026-10545-0.
6. Chen A, Liao S, Cheng M, Ma K, Wu L, Lai Y, et al. Spatiotemporal transcriptomic atlas of

mouse organogenesis using DNA nanoball-patterned arrays. Cell. 2022;185 10:1777-92.e21; doi:10.1016/j.cell.2022.04.003.

7. Shi Y, Wang M, Mi D, Lu T, Wang B, Dong H, et al. Mouse and human share conserved transcriptional programs for interneuron development. Science. 2021;374 6573:eabj6641; doi:10.1126/science.abj6641.

8. Webb S, Rose A, Xu C, Steele L, Kuri MA, Stephenson E, et al. An integrated single-cell and spatial omics atlas of human prenatal development. bioRxiv. 2026:2026.03.30.714220; doi:10.64898/2026.03.30.714220.

9. Jagadeesh KA, Dey KK, Montoro DT, Mohan R, Gazal S, Engreitz JM, et al. Identifying disease-critical cell types and cellular processes by integrating single-cell RNA-sequencing and human genetics. Nat Genet. 2022;54 10:1479-92; doi:10.1038/s41588-022-01187-9.

10. Wang J, Ye F, Chai H, Jiang Y, Wang T, Ran X, et al. Advances and applications in single-cell and spatial genomics. Science China Life Sciences. 2025;68 5:1226-82; doi:10.1007/s11427-024-2770-x.

Personal Cover

Click here to access/download;Personal Cover;Cover letter.docx

Dear Editor,

We are pleased to submit our invited manuscript titled **"Charting human organogenesis across the Carnegie stages from a whole-embryo perspective"** for consideration as a Perspective in ***GigaScience***.

As kindly invited by the editorial team, this piece provides a comprehensive perspective on the significance and future implications of our upcoming research article, *"Spatiotemporal transcriptome atlas of human embryos after gastrulation",* which is currently in the final production stages and will soon be published in ***Nature*** (DOI: 10.1038/s41586-026-10545-0).

In this invited commentary, we contextualize the importance of the Carnegie stages and discuss the critical shift in developmental biology from fragmented, organ-specific datasets toward integrated, whole-embryo spatiotemporal frameworks. While our upcoming ***Nature*** paper presents the dynamic molecular map itself, this ***GigaScience*** piece expands on what such a work means for the broader scientific community. We explore how restoring spatial and anatomical context allows for a deeper mechanistic understanding of early cardiac patterning, brain regionalization, tissue-specific vulnerabilities to prenatal infection, and spatially resolved allelic imbalance.

We confirm that this manuscript is original, has not been published elsewhere, and is not under consideration by another journal. All authors have reviewed and approved the final version of the manuscript and agree to its submission to ***GigaScience***.

Thank you for your time, the kind invitation to contribute to your journal, and your consideration of our work. We look forward to your response.

Sincerely,

Guolian Ding, MD, PhD
Hefeng Huang, MD, FRCOG
Jiexue Pan,MD, PhD

Institute of Reproduction and DevelopmentObstetrics and Gynecology Hospital,
Fudan University Shanghai, 200032, China

Email: dingguolian@fudan.edu.cn
